# Evidence for surprise minimization over value maximization in choice behavior

**DOI:** 10.1038/srep16575

**Published:** 2015-11-13

**Authors:** Philipp Schwartenbeck, Thomas H. B. FitzGerald, Christoph Mathys, Ray Dolan, Martin Kronbichler, Karl Friston

**Affiliations:** 1 The Wellcome Trust Centre for Neuroimaging, UCL, 12 Queen Square, London, WC1N 3BG, UK; 2 Centre for Cognitive Neuroscience, University of Salzburg, Salzburg, Austria; 3 Neuroscience Institute, Christian-Doppler-Klinik, Paracelsus Medical University Salzburg, Salzburg, Austria; 4 Max Planck University College London Centre for Computational Psychiatry and Ageing Research, London WC1B 5EH, UK

## Abstract

Classical economic models are predicated on the idea that the ultimate aim of choice is to maximize utility or reward. In contrast, an alternative perspective highlights the fact that adaptive behavior requires agents’ to model their environment and minimize surprise about the states they frequent. We propose that choice behavior can be more accurately accounted for by surprise minimization compared to reward or utility maximization alone. Minimizing surprise makes a prediction at variance with expected utility models; namely, that in addition to attaining valuable states, agents attempt to maximize the entropy over outcomes and thus ‘keep their options open’. We tested this prediction using a simple binary choice paradigm and show that human decision-making is better explained by surprise minimization compared to utility maximization. Furthermore, we replicated this entropy-seeking behavior in a control task with no explicit utilities. These findings highlight a limitation of purely economic motivations in explaining choice behavior and instead emphasize the importance of belief-based motivations.

Modern economic models of choice, dating to the 1950s, suggest that the aim of human decision-making is to maximize ‘utility’, a quantity tied to notions of pleasure and reward^1^,^2^,^3^. More recently a different line of reasoning has stressed the notion of homeostasis (or allostasis), recognizing that agents maintain their states within certain bounds to persist in the face of a changing world^4^,^5^,^6^. From an information-theoretic perspective^7^ this implies that agents have to optimize a model of their environment and, given that model, minimize surprise about the states they find themselves in. This is equivalent to maximizing the evidence for their model of the world (because, negative surprise is Bayesian model evidence). Based on this view, belief- as opposed to reward-based formulations of choice behavior are attracting interest^8^,^9^,^10^,^11^.

For a purely passive agent, surprise can be minimized through learning and inference (changing an internal model to make better predictions). However, embodied agents are capable of acting on the world, enabling them to minimize surprise through (epistemic or pragmatic) actions and select the outcomes they expect (i.e., are the least surprising). This notion is at the heart of active inference, which provides an increasingly influential account of action and choice behavior^8^,^12^,^13^,^14^,^15^. Active inference rests on the assumption that the brain is a Bayesian inference machine that encodes beliefs about the world, rendering cognition probabilistic. Here the concept of reward is replaced by prior expectations about occupying different states, where ‘desired’ or preferred outcomes are more likely, given an agents’ beliefs. An active inference agent is compelled to minimize surprise by realizing preferred outcomes, where surprise can be quantified by the Kullback-Leibler divergence (relative entropy) between the agent’s prior preferences and posterior beliefs about likely outcomes given current observations^11^,^16^,^17^.

A key difference between belief-based and economic models of choice is the former predicts behavior should be sensitive to both reward and information or entropy gain, whereas the latter predict that humans are exclusively sensitive to the rewarding value of outcomes:


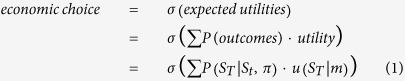



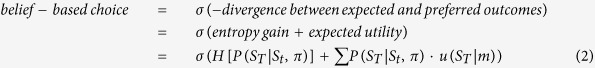


Where 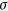 denotes a softmax choice-rule, 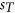 refers to final outcomes, 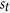 to a current state, *π* to a policy (i.e., a sequence of control states 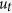, which determine the likelihood over final outcome states 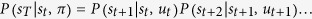). Here, 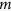 denotes an agent’s model—that includes prior preferences or utilities (i.e., a probability distribution over final states that is determined by the agent’s expectations or beliefs about the states it aspires to; see methods for details).

Each policy prescribes a unique sequence of probability transitions given the control states chosen by the agent, and the agent has to choose among (allowable) policies. Note that these sequential policies are more general than classical state-action policies. Thus, classical utility-based models predict that agents maximize expected utility based on their preferences, whereas active inference predicts that agents will maximize both utility and entropy, which can be understood intuitively as ‘keeping ones options open’ or ‘planning to be surprised’^18^ (see Fig. 1 for an illustration). It is important to appreciate that entropy is not equivalent to classical notions of exploration or novelty seeking, since its aim is *not* to decrease uncertainty over outcomes but rather to keep options open^19^,^20^. In short, active inference predicts that purposeful behavior will be governed by both entropy and utility maximization, where the relative contribution of the two depends on the relative value of outcomes^21^. Our experimental paradigm was designed to test this prediction.

## Results

We created a novel paradigm to test for entropy-seeking in a group of healthy subjects (*n* = 20). Participants had to decide between two options, each containing a lottery varying in the type and number of snacks (Fig. 2). Subjects were told that, at the end of the experiment, they would receive one of their choices (selected at random). The number of snacks comprising a lottery determined the entropy over snacks a subject could win. If subjects selected a lottery with only one snack (e.g., a chocolate bar), then there is full certainty about the outcome (i.e., no information gain). If there were several snacks in the selection, then any of the snacks could be chosen with equal probability 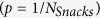. We ensured that subjects cared about which snack they could win by asking them to refrain from eating for at least 3.5 hours before testing. Furthermore, we did not use snacks that subjects either strongly liked or disliked, to ensure a meaningful decision, and to ensure that the sequence of trials produced roughly orthogonal variations in entropy and utility.

Crucially, in this task entropy over outcomes can be understood as the potential to secure different snacks. Given that, in our design, all outcomes were equally probable; this is equivalent to the number of snacks in an option (i.e., the cardinality of the outcome space). Importantly, minimizing the (KL) divergence between predicted and preferred states rests on choices with high entropy over outcome states, thus ‘keeping options open’, even if the expected utility is exactly the same.

This task was ideally suited to compare a belief-based and a purely economic formulation of choice. If subjects solely maximized reward or utility, then their choices would be determined exclusively by trying to secure preferred snacks. Thus, subjects would always choose the option with highest expected utility; i.e., the best combination of utility and likelihood of snacks in the two options. Conversely, if subjects minimize surprise (KL divergence), they will also try to maximize entropy over outcomes; in other words, the number of distinct snacks they could win (see equation 4 in methods for details). It is known that in these types of choice paradigms risk, defined as variance over the utilities of outcomes, plays an important role in predicting choices, where some subjects will exhibit risk-seeking and others risk-avoiding behavior^1^,^22^. Therefore, we included risk as an additional explanatory variable, when comparing economic to belief-based models. To assess whether entropy was indeed a significant factor in choice behavior, we performed a linear regression to predict subject’s decisions on each trial using the difference in expected utility (based on individual rating of the snacks), risk and entropy of the two options.

We found that both expected utility and entropy were significant determinants of choice behavior (one-sample test: 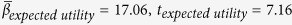, 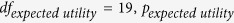 <0.001;  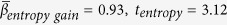, 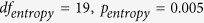; see Fig. 3A and Table 1 for a list of the regression coefficients). This means that subjects displayed a tendency to maximize both expected utility and the entropy over outcomes. Given that half the trials presented options of similar utility but different entropy—which were central for testing our hypothesis—we repeated the above analysis for these trials only. Again, we found that the two factors were necessary to explain choice behavior (one-sample test: 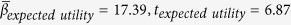, 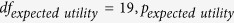 < 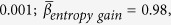 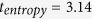, 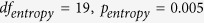).

These results are consistent with a tendency to maximize entropy in addition to maximizing expected utility (while seeking or avoiding risk). Furthermore, using the Bayesian Information Criterion (BIC) to approximate the evidence for the linear regression models, we compared a model including expected utility and risk to one containing expected utility, risk and entropy. In keeping with active inference, random effects Bayesian model comparison^23^ provided strong evidence for the model that included entropy (exceedance probability *φ* = 0.99; exceedance probability for manipulated trials only *φ* = 0.92). Random effects Bayesian model comparison is a variational Bayesian method that treats each model as random variable and estimates the parameters of a Dirichlet distribution describing the probability that each model generated the data (see^23^ for details). Crucially, this (random effects) method is more robust to outliers than classical fixed effects metrics.

Further, we performed a more formal (model-based) analysis, using a framework for modelling behavior on Markov Decision Processes^8^,^15^, allowing for a comparison between KL-control and utility maximization within the framework of active inference. This framework has been successfully applied to various problems in decision-making^14^,^24^,^25^. Furthermore, it is ideally suited to compare (belief-based) active inference formulations of choice to expected utility theory: by predicting trial-by-trial choice-probabilities that can be compared with observed behavior (see Fig 4 for an example).

In our active inference scheme, a key role is played by the precision (i.e. confidence or *γ*) of beliefs about policies or choices, which controls the stochasticity of behavior^15^,^24^. Precision accounts for an agent’s sensitivity to its beliefs about choosing an option—as mandated by either minimizing surprise (active inference) or maximizing expected utility (economic models). Precision therefore plays the role of a sensitivity or inverse temperature parameter in conventional softmax choice rules. Crucially, active inference suggests that subjects optimize their precision as well as their choices, where each subject has prior beliefs about precision (these prior beliefs are parameterized with a gamma distribution). Using constrained maximum likelihood, we estimated each subject’s prior precision as well as their preferences for snacks, and compared a model based on KL-control to a model based on expected utility (see methods for details). To test whether differences in behavior were indeed accounted for by differences in their prior distribution over precision, we evaluated the evidence (BIC) for both schemes when allowing prior precision (alpha or the scale parameter of a gamma distribution with beta = 1) to be a free parameter (included in the parameter count of the BIC scores) and when using a fixed prior over precision for all subjects (with parameters alpha = 4 and beta = 1). Precision updates during choices are discussed in detail elsewhere^8^,^15^,^24^: briefly, when casting choice behavior as active inference, precision (i.e., confidence) becomes a hidden variable that has to be inferred on a trial-by-trial basis. This implies that precision ceases to be an *ad hoc* parameter (such as inverse temperature in conventional models) but acquires a context-sensitive Bayes optimal solution. However, in our one-shot (single trial) snack lottery, precision updates are precluded and choices depend only on prior precision. This prior precision models the stochasticity of each subject’s behavior.

As in the previous analysis, Bayesian model comparison provided strong evidence in favor of an active inference model of choice behavior that included subject-specific prior precision (exceedance probability 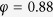; 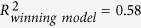; see Fig 3B). Furthermore, we found that the maximum likelihood estimators of subject’s preferences in the winning model correlated highly with their preference rating of the snacks (*r* = 0.90), providing a reassuring validation of our estimation scheme.

In summary, we performed two analyses of behavior in our task, one based on an explicit measure of subject’s preferences (or subjective utility)—as indicated in a rating of the snacks after the experiment—and the other based on an implicit measure, obtained by estimating preferences using observed behavior. We used the explicit measure in a simple linear regression analysis, testing for effects of expected utility, risk and entropy on behavior. The implicit estimates were obtained via (constrained) maximum-likelihood estimation in a computational (Markov decision process) model of the task, which compared active inference to expected utility theory. In brief, we found compelling evidence for surprise minimization in choice behavior in both analyses, suggesting that behavior is governed by both expected utility and entropy maximization. Furthermore, we found a strong relationship between the explicit and implicit measures of subject’s preferences.

**Figure 1:**
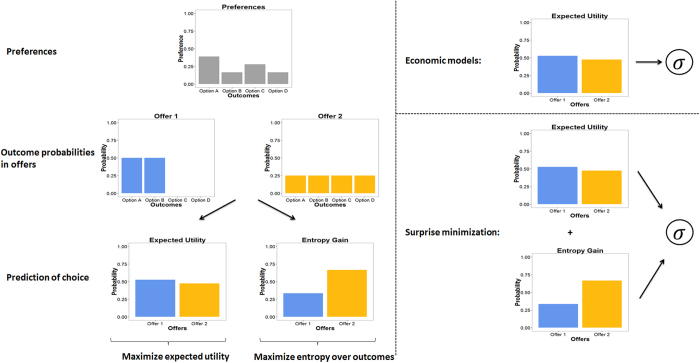
Difference between classical economic models of choice and active inference. On the left, an example of two offers is illustrated, where offer 1 (left, blue bars—middle panel) contains two possible outcomes and offer 2 (right, yellow bars—middle panel) contains four different outcomes with equal probability. Based on a given set of preferences (grey bars—top panel), the expected utilities of offer 1 and 2 are almost identical, whereas the entropy over outcomes is higher in offer 2. On the right, choice tendencies under classical economic models and under surprise minimization are illustrated, where 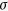 denotes a (softmax-) choice rule. While classical economic models predict that behaviour will be governed by maximising expected utility alone (top right), active inference predicts that agents are additionally compelled to maximize entropy over outcomes (bottom right).

**Figure 2:**
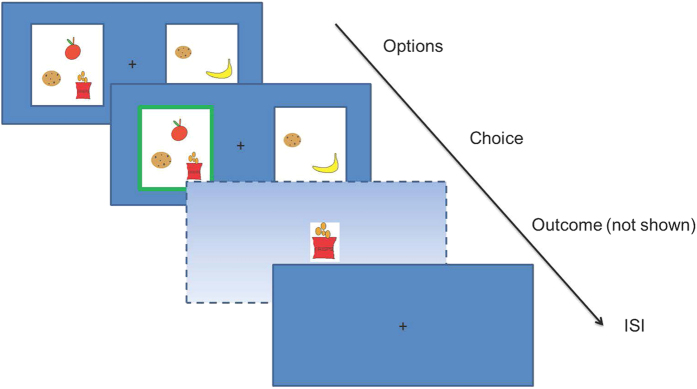
Task. Subjects had to choose between two offers containing between one and four different snacks. Snacks could only appear once in each option, but the same could appear in each offer. Subjects knew that at each trial one of the snacks of their chosen offer would be selected randomly (without knowing which one) and at the end of the experiment they would receive one of the selected snacks. Thus, the number of snacks in the chosen option determined the probability of winning one of snacks. In this example, subjects had a 1/3-chance of winning a cookie in the left and a 1/4 –chance of winning a cookie in the right option.

**Figure 3:**
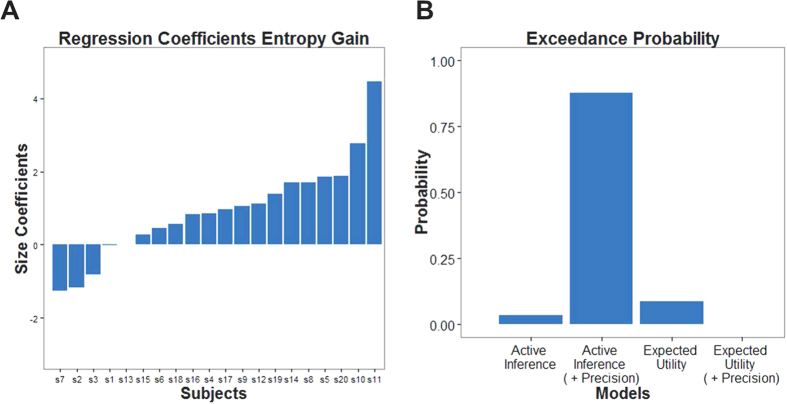
Model-based analysis: (**A**) Individual regression coefficients for entropy as obtained in a linear regression predicting choices. (**B**) Exceedance probabilities in random-effects Bayesian model comparison provide evidence for an active inference model (with individually estimated prior precision) over expected utility theory and equivalent models with a fixed precision. Note that these exceedance probabilities are computed with respect to the BIC-scores of the models and thus adhere to an accuracy-complexity trade-off.

**Figure 4:**
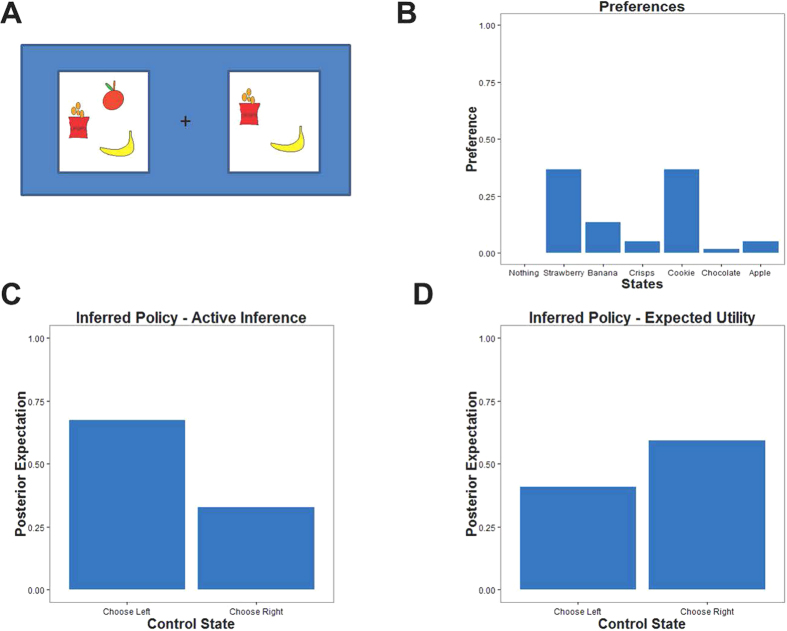
Comparing active inference and value maximization predictions of choice. (**A**) illustrates a trial where two options differ in number of snacks but only marginally in their expected utility based on a subject’s individual preference of snacks (displayed in (**B**)). Active inference predicts a choice tendency towards the option with more snacks (shown in (**C**)) while value maximization predicts a choice preference for the option with (marginally) higher expected utility (shown in (**D**)).

Surprise minimization makes predictions about choice behavior that are at variance with classical economic theories, when the expected utilities of two options are very similar but the entropies over outcomes differ. In these cases, expected utility theory predicts that the two options would be chosen with the same probability, whereas surprise minimization predicts a choice bias for the option that has the higher entropy (as displayed in Figs 1 and 4). Therefore, one should expect to find entropy-seeking behavior when expected utility theory predicts a (close to) random choice. To test this, we looked at these trials specifically and asked whether subjects prefer higher entropy options. We used the two winning models based on active inference and expected utility theory, respectively, and tested their predictions in trials where the former model predicted a clear preference for one option but the latter predicted a (close to) random choice or even a preference for the other option (see methods for details). We found that in those trials, subjects displayed a high proportion (75%) of responses that were in line with surprise minimization rather than expected utility maximization, significantly larger than 50% on the group level (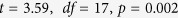). This suggests that in trials with similar expected utilities, subjects adhered to surprise minimization; i.e., maximizing the entropy over outcomes, rather than economic models of decision-making.

**Figure 5:**
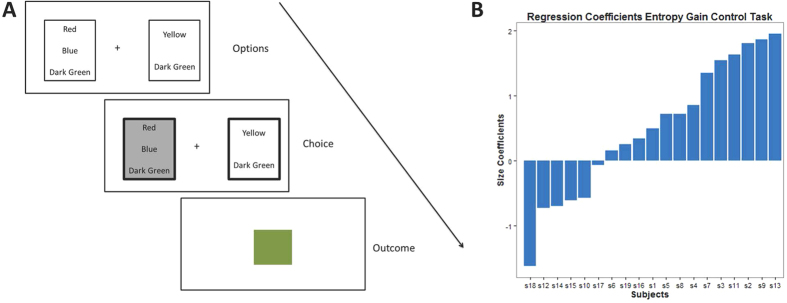
Control task. (**A**) In a control experiment, subjects were asked to make a choice between options containing colors rather than snacks. After every trial, subjects could see one of the colors of their chosen option on the screen in form of a square. Crucially, the number and type of colors differed between the two options, containing between one and four different colors. (**B**) In a simple regression analysis, we found a substantial choice bias to choose options that maximize the entropy over outcomes as predicted by surprise minimization.

Finally, active inference is also applicable to choice behavior in the absence of utilities over outcomes. In other words, subjects should also try to maximize the entropy over outcomes that have no explicit utility. We tested this prediction in an additional control experiment, where snacks were replaced by different colors (see Fig 5A for an illustration). In this task, 19 healthy subjects had to choose between two options that contained between one and four different colors (out of six colors in total – using colors that subjects neither strongly liked nor disliked). After having chosen one of the two options, subjects were subsequently presented with one of the colors in their chosen option (each with equal probability), in form of a square on the screen. Crucially, while subjects may form implicit preferences for certain colors, this experiment lies outside the domain of classical economic theory and utility maximization given that the outcomes were purely perceptual. Still, surprise minimization predicts that subjects should prefer options with higher entropy over outcomes, i.e. with more colors.

To test this hypothesis, we used a regression model with observed choices (+1 for left and −1 for right) as the response variable and the difference in entropy between the right and left options as an explanatory variable. To ensure our inference was not confounded by any implicit preference for specific colours, we used colour as a confound (+1 for present in the right option, −1 for present in the left option and 0 if present in both or neither).

As in the main experiment, we used the standard summary statistic approach to random effects analysis, using a one tailed t-test on (subject specific) regression coefficients reporting entropy effects (shown in Fig 5B). We confirmed the anticipated (positive) effect for entropy (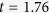, 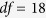, 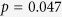). The significance of this effect increased when including the entropy times colour interaction as a cofound (i.e., the implicit value of a colour was allowed to vary with the number of choice items it was presented with; 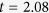, 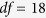, 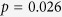). Furthermore, we again found a high correlation between implicit (estimated) preferences for the colors and subjects’ explicit preferences from ratings after the experiment (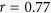). In six subjects this correlation was remarkably high (greater than 0.9).

## Discussion

Our findings provide evidence for a belief-based formulation of human choice behavior that is motivated by minimizing surprise rather that maximizing utility alone. We designed a task in which we could test a key prediction of active inference that is at variance with expected utility theory. Minimizing surprise means that our choices should maximize both the utility and the entropy over outcomes, which can be understood as expected surprise or ‘keeping ones options open’. In other words, given the choice between a single outcome and multiple outcomes with the same utility, we prefer the latter. Note the apparent paradox of minimizing surprise about states we visit and expecting ourselves to maximize entropy. This is paradoxical because entropy is average surprise. The apparent paradox is resolved by noting that entropy is also average information, which has previously been associated with novelty, information gain or epistemic value^26^. Put simply, our results indicate that we expect ourselves to maximize utility, while keeping our options open. Our results thus provide evidence that choice behavior maximizes both expected utility and epistemic value – as opposed to just expected utility.

Our paradigm included trials in which the expected utility of two options containing snacks were very similar but one of the options provided higher entropy over outcomes. Given that we assigned equal probabilities to all outcomes (snacks), entropy over outcomes corresponds to the number of snacks offered in an option. The only way to change entropy over outcomes is to have a different number of *different* snacks in the two options. Therefore, it was impossible to design trials that had identical expected utility. However, we ensured that the expected utilities of options were very similar by only selecting snacks that had similar utilities (based on their pre-experiment rating and by excluding snacks that were explicitly liked or disliked). Furthermore, we ensured half of the trials had similar expected utilities but different entropies. These trials were the most interesting ones from the perspective of our hypothesis: expected utility theory would predict a roughly random choice in these trials, whereas active inference predicts a choice bias towards the option with higher entropy or number of different (but equally preferred) snacks. Additionally, we carried out a control task, in which subjects ‘played for’ different colors—as opposed to snacks they could eat, thus removing (explicit) utility as a determinant of choice behavior. We again found that subjects showed a substantial bias to maximize the entropy over outcomes, indicating that the concept of surprise minimization also applies to choice behavior beyond the domain of behavioral economics.

A natural question arising from these considerations concerns the developmental and evolutionary advantage of maximizing the entropy over outcomes—in addition to maximizing utility or value. Why should we care about entropy at all and not choose the option that has highest utility or reward? Generally, our findings speak to a probabilistic formulation of cognition and brain function. Put simply, if the brain is a Bayesian inference machine^27^,^28^,^29^,^30^, choice behavior is an active inference process that minimizes surprise. In turn, this requires choices that minimize the difference between expected and preferred (unsurprising) outcomes, leading to risk-sensitive or KL control. This means that choices should be sensitive to both expected utility and entropy over outcomes, not expected utility alone. Maximizing the entropy over outcomes and thus ‘keeping options open’ may have adaptive advantages that endow choice behavior with flexibility. This may be particularly important when different options or policies have a similar expected utility. Furthermore, not committing to a specific outcome may also be important for ensuring a continuous updating of an agent’s beliefs in a changing environment. Finally, maximizing the entropy over outcomes complies with the principle of maximum-entropy^31^, which says that the least biased inference results from adopting the prior that has maximal entropy.

Crucially, our findings contextualize classical models based on expected utility maximization^1^,^2^,^3^. A choice bias for maximizing entropy over outcomes may be particularly important for understanding seemingly ‘irrational’ behavior, such as not always choosing the most valuable option. Some previous accounts have assumed similar biases in choice, such as self-signaling or ‘diagnostic utility’ of outcomes in addition to classical (economic) utilities^32^ or curiosity-based accounts of decision making that maximize Bayesian surprise^33^,^34^. A rigorous formalism that casts choice behavior as minimizing surprise, however, has not been tested so far.

It has been proposed that modern economic models of choice can be characterized on a continuum between purely value-based accounts, where the brain is assumed to compute and represent the values of all options, and purely comparison based accounts, such that the utilities or values of different outcomes are determined through comparison with other available outcomes^35^. In the model we presented, surprise minimization does not make strong assumptions about how these utilities are formed. The crucial prediction of surprise minimization is that agents encode expectations about future states, which reflect their preferences under a given model of the environment. However, it should be noted that the ‘sum-to-one’ constraint of beliefs over outcomes makes it impossible to consider the preference for one outcome in isolation from the others. This suggests a close relationship between surprise minimization and comparison-based approaches to choice behavior, which highlight the relative nature of utility representations or even sensory experiences (for a review see^35^). How these expectations are acquired (or change in time via learning) remains a key question. Previous accounts, in the context of ‘stochastic expected utility theory’ have assumed random fluctuations in choice (over utilities, model parameters or actual choice behavior) that may explain why subjects do not always choose the option with highest expected utility^36^,^37^. In contrast, surprise minimization suggests that these apparent ‘noisy decisions’ may in fact reflect an additional choice bias; namely, maximizing the entropy over outcomes. Furthermore, keeping options open may also have an important relationship to so called ‘fast-and-frugal-heuristics’ and bounded rationality^38^. Maximizing entropy may be particularly advantageous if an agent is unable to assess the expected utilities of options under given policies due to time or resource constraints. Bounded rationality in the context of surprise minimization and, more specifically, the relationship between entropy and expected utility maximization is another important field for future research and may relate to ideas about Bayesian model selection and averaging in the brain, as well as restricting the model space for subsequent inference on parameters or hidden variables^39^.

We do not argue that entropy gain cannot be accommodated within a utilitarian model, as in the example of exploration gain or novelty bonuses^40^. However, casting choice as surprise minimization mandates such preferences, as opposed to introducing *ad hoc* variables to describe choice tendencies. In this regard, our framework offers a more formal basis, contextualizing observations such as Pavlovian biases or perceptual preferences (e.g., for more items on the screen). Furthermore, active inference is equipped with a process theory; i.e., variational message passing, which offers a biologically plausible model of how the requisite computations might be implemented in the brain. The notion that entropy or information gain can be integrated with expected utility in a principled and normative fashion—to prescribe behavior—suggests that it should also operate in the absence of any preferences or subjective utilities. This observation suggests that one can predict behavior in the absence of utility, as illustrated in our control (playing for colors) experiment. Interestingly, the active inference formulation was inspired by accounts of information foraging in visual searches, specifically, the resolution of uncertainty afforded by saccadic eye movements to salient locations in the visual field^41^. Formally, the entropy in this context can be associated with the relative entropy or Bayesian surprise^42^ associated with a sampling of the visual field. If this reasoning holds, it should be possible to use the active inference scheme described above to model saccadic eye movements; provided they can be modelled in a discrete state space. We will pursue this in future work.

In conclusion, we provide evidence for a belief-based (active inference) formulation of choice, based on surprise minimization, as opposed to a classical economic treatment of decision-making as maximizing value or utility. This evidence suggests that agents maximize both expected utility and entropy. These considerations offer a new perspective on cognition and brain function as Bayesian inference processes and provide a new set of tools for understanding the mind as a (generative) model of the environment. These tools may provide important insights into how agents ‘make sense out of the world’ and how certain aberrancies in these processes may induce psychopathological behavior^43^,^44^,^45^,^46^.

## Methods

### Subjects

20 subjects (16 female) participated the first (main) study. In addition, three incomplete data-sets were collected (post-experiment ratings not recorded) which were excluded from the analysis. All subjects were university students from the University of Salzburg (Austria) and had a mean age of 24.8 (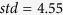, 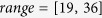). Written informed consent was acquired from all participants and they were reimbursed with €10 for taking part in the study. In the second (control) study, 19 subjects (15 female) participated, which were recruited from the same subject pool. These subjects had a mean age of 22.9 (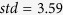, 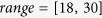) and were reimbursed with €15 for their participation. All experimental protocols were approved by the local ethics committee of the University of Salzburg. All methods were carried out in accordance with the approved guidelines.

### Task (main study)

Subjects underwent 1.5 hours of testing in a behavioral testing room at the University of Salzburg. After obtaining written informed consent and reading an instruction sheet, subjects reported when they had most recently eaten (mean: 4.5 hours) and how hungry they felt—on a scale from 1 to 11 (median: 8). Since the experiment involved playing a lottery for real snacks, we ensured that subjects would be sufficiently hungry to care about their win by instructing them not to eat for at least 3.5 hours prior to the experiment.

Subsequently, subjects rated a total of 18 snacks (each snack was rated three times), which were placed next to the screen (and could be seen by subjects). For the following experiment, we used the six snacks in the middle of the ranking. This ensured that subjects did not play for snacks they disliked or preferred—and therefore decisions were sufficiently deliberative. Importantly, we only used snacks that are known to have little or no variance in their taste (such as particular brands of chocolate bars, cookies or crisps), as opposed to snacks with indefinite properties (such as fruit). This ensured that subjects had a precise idea about the snack they might win at the end of the experiment.

After the initial rating, subjects played three sessions consisting of 100 trials (~15 minutes). Each trial involved a choice between two options or ‘lotteries’ displayed on the left and right side of the screen. The two options differed in number and type of snacks offered to the subject and could contain between one and four different snacks. Subjects had to decide whether they wanted to choose the left or right lottery, knowing that at the end of the experiment one of their choices would be selected randomly and the chosen lottery would be played out. The term ‘lottery’ here simply means that the subjects would win one of the snacks contained in the chosen option, each with equal probability 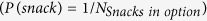. For example, if their selected choice was an option containing only one snack there was full certainty over their win, whereas an option with four snacks gave a ¼ -chance of winning one of them. To ensure that we had enough trials differentiating between an expected utility and KL-control prediction of choices (see Fig 4 in main text), we designed half the trials to have a similar expected utility but different entropy gains; i.e., a different number of snacks available in the two options. In these trials, we ensured the difference in expected utility of the two offers did not exceed a fifth of the difference between the least and most preferred snack, based on the subject’s rating prior to the experiment.

After completing the third session, subjects were presented with their win and, subsequently, rated the snacks again to test whether their preferences had changed over the course of the experiment. We found that ratings indeed became more clearly defined as indicated by a weak but significant correlation between prior- and post-experiment ratings (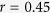, 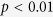). Thus, we used the post-experiment rating for the subsequent regression analysis to estimate subject’s individual differences in expected utility and risk. This assumes that the post-experiment rating reflected subject’s preferences more precisely that the pre-experiment rating. This assumption was confirmed by a substantially higher correlation between modelled preferences in our constrained maximum likelihood estimation, based on subject’s behavior, and post-experiment ratings compared to pre-experiment ratings (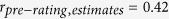, 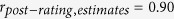). We therefore concluded that the post-experiment ratings reflected true preferences, while avoiding any confounds due to a preference-change over the course of the experiment.

### Task (control study)

The control task closely resembled the original task but presented different colors as opposed to different snacks in the two options (see Fig 5A for an illustration). After an initial rating of colors, we selected six colors that were neither explicitly preferred nor disliked. Subsequently, Subjects played three sessions consisting of 60 trials (each lasting for about 15 minutes). The first 50 trials of the first session were treated as training trials, because—in contrast to the main study—subjects had to establish an association between the colors and outcomes (and possibly develop implicit preferences). The two options in each trial could contain between one and four different colors. Subjects had to make a decision after every trial and were subsequently presented with one of the colors of their chosen option in form of a square (displayed for four seconds). The outcome of a trial was again sampled from a uniform distribution conditioned on the number of different colors of the chosen option. After completing the experiment, subjects were asked to rate the colors again.

### Behavioral analysis and modelling

All statistical analysis was performed using MATLAB version R2012a. Initially, we conducted a regression analysis to assess whether choices (between the two options) would be predicted by expected utility, risk and entropy gain. To do so, we calculated the difference in expected utility, risk and entropy gain on a trial-by-trial basis between the two options and tested which of these differences predicted choices. For each option, expected utility was defined as the sum of the values of the snacks multiplied with their respective probability: 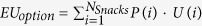, where 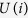 was obtained individually from subject’s preference-rating. Risk was defined as variance over the utilities in the two options: 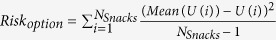-. Finally, entropy gain was operationalized as entropy over outcome-probabilities: 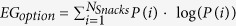. If any of these factors influenced choice behaviour, then the respective difference in this measure between the two options should be a significant predictor of observed trial-by-trial choices. Individual regression coefficients for each subject are displayed in Table 1. To assess overall significance, we performed a second-level analysis (one-sample t-test) on the regression coefficients of expected utility and entropy gain.

Subsequently, we performed model comparisons for models with and without entropy gain. We computed model accuracy, i.e. the log-likelihoods of the observed responses given a general linear (regression) model either with expected utility and risk alone or with entropy gain as additional factor. Based on these log-likelihoods, we calculated the Bayesian Information Criterion (BIC, see Table 2) for each of the two models—penalizing for the number of parameters used—and performed random effects Bayesian model comparison^23^ based on these values using the function spm_BMS of SPM12b (Wellcome Trust Centre for Neuroimaging, London, UK, http://www.fil.ion.ucl.ac.uk/spm).

Subsequently, we performed a model comparison based on an active inference formulation of behavior, casting the task as a Markov Decision Process (MDP) described in more detail elsewhere^8^. We specified the routine implemented in the spm_MDP_offer.m routine of the DEM toolbox of SPM12 to provide trial-specific probabilities of choice that could be compared with observed behavior. Crucially, this routine rests on a KL-control formulation of choice, such that agents try to minimize the difference or KL-divergence between ‘likely’ and ‘desired’ outcomes:


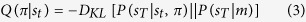


Equation 3 states that the value of a policy 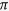 at a particular state 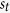 is determined by the (negative) difference between a probability distribution over outcome states 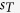
given the current state and that policy and a probability distribution over outcome states given an agent’s model; i.e., where an agent believes it will end up in. These two distributions reflect what an agent believes is likely to happen and what it expects to happen, given its preferences over outcomes. More formally, these distributions refer to posterior beliefs about outcomes conditioned on beliefs about its current state (and policy) and prior beliefs about outcomes. By minimizing this difference, agents select policies that bring controlled outcomes closer to preferred ones. Crucially, this KL-divergence can be rewritten as


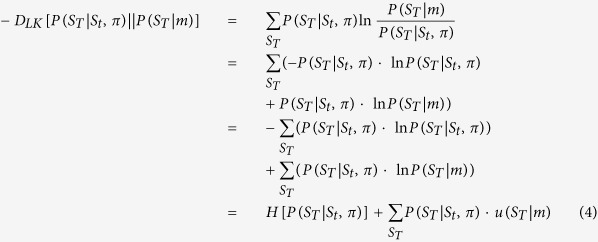


Equation 4 shows that minimizing the KL-divergence implies maximizing the entropy over outcomes and the sum of the utility of outcomes multiplied with their probability. The first term reflects entropy gain, and can be maximized if agents select options that ensure a wide probability distribution over outcomes. The second term reflects standard expected utility based on an agent’s beliefs where it should end up in.

Crucially, one can compare the predictions from this formulation to standard expected utility theory by ‘switching off’ the entropy term in equation 4. Thus, we used a KL-control and expected utility version of this routine to predict choice behavior and compared their respective model evidences (displayed in Table 3), while we estimated individual priors on precision and preferences for the snacks using constrained maximum likelihood estimation (alpha constrained between 2 and 8 and preferences constrained to lie between −10 and 10) (see Table 4 for individual parameter estimates). Crucially, therefore, the estimation of free parameters was unrelated to the specifics of the computational scheme that was used to predict subjects’ preferences for one of the two options, where the individual prior on precision can be thought of as an inverse temperature parameter in classical softmax choice rules and the preferences for snacks as classical utility functions over outcomes.

Furthermore, in active inference not just beliefs about behavior have to be optimized but also the certainty or precision of these beliefs has a (Bayes) optimal solution. Choices, therefore, result from beliefs about behavior multiplied with precision 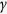:


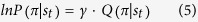


The important implications of precision for behavior and its putative neuronal underpinnings are discussed elsewhere^15^,^24^. In brief, precision simply plays the role of an inverse temperature making subjects more or less sensitive for the value of choosing an option. Since precision potentially also plays an important role in this context, we compared a version of the KL-control (and expected utility scheme) in which precision was optimized and in which precision was fixed across-trials (to a standard value of four for all subjects: 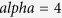). Bayesian model comparison provided strong evidence in favor of an active inference model of choice in which precision was estimated for each subject individually (exceedance probability 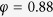).

To analyze behavior in trials where surprise minimization predicted a preference for one of the two options (due to a difference in entropy over outcomes) whereas expected utility maximization predicted a similar bias for both options or even a preference for the opposite option (as displayed in Fig. 4), we selected trials in which the active inference scheme predicted a probability of acceptance for one of the two options of 70% or above whereas the expected utility scheme predicted a propensity to accept of 55% or lower (for that option). We then computed the relative proportion for each subject to select the option favored by active inference, but not by expected utility theory. Two subjects had to be excluded from this analysis because for them the predictions of the model predictions did not differ enough, such that no trials met the criteria explained above.

The analysis of the control study used the same linear regression approach as adopted in the main study; however, the within subject model included (confounding) explanatory variables accounting for any implicit effects of each color in the available options (as opposed to effects based upon explicit utility ratings in the first study). To avoid ceiling effects, only trials with less than four options were included in the regression.

## Additional Information

**How to cite this article**: Schwartenbeck, P. *et al.* Evidence for surprise minimization over value maximization in choice behavior. *Sci. Rep.* **5**, 16575; doi: 10.1038/srep16575 (2015).

## Figures and Tables

**Table 1:** Individual regression coefficients in the linear regression for the difference in expected utility, risk and entropy in the two options (statistical significance indicated with an asterisk).

Subject	Expected utility	Risk	Entropy gain
1	6.33**	−2.83*	−0.02
2	8.36**	10.47**	−1.18**
3	8.14**	−7.49	−0.82**
4	16.96**	−10.28**	0.86**
5	14.94**	5.63	1.87**
6	9.31**	−13.47**	0.46*
7	0.47	8.59**	−1.27**
8	24.04**	19.66**	1.71**
9	27.63**	−15.88	1.05**
10	43.80**	15.02	2.78**
11	6.89**	4.16	4.46**
12	15.97**	−39.49**	1.12**
13	17.75**	10.84**	0.01
14	19.06**	6.22*	1.69**
15	18.78**	−36.16**	0.28
16	10.82**	−7.33**	0.83**
17	9.11**	−45.29*	0.97**
18	30.61**	6.47	0.57**
19	33.43**	−20.44*	1.39**
20	18.76**	15.06**	1.88**

Positive coefficients indicate a positive choice bias (seeking), whereas negative coefficients indicate avoidance. Thus, all 20 subjects tried to maximize expected utility, 10 out of 20 subjects were risk-seeking and 16 out of 20 maximized entropy gain.

**Table 2:** Individual BIC scores for the general linear models with and without entropy gain.

Subject	Without Entropy Gain	With Entropy Gain
1	−263.65	−269.33
2	−349.92	−326.58
3	−310.91	−303.47
4	−196.36	−192.60
5	−257.96	−219.29
6	−320.01	−320.78
7	−425.83	−391.45
8	−222.29	−201.65
9	−199.72	−193.29
10	−264.21	−206.91
11	−358.05	−185.59
12	−282.37	−268.50
13	−197.28	−202.96
14	−183.95	−167.31
15	−276.33	−280.31
16	−263.10	−258.83
17	−373.01	−354.93
18	−321.27	−321.06
19	−239.36	−218.97
20	−158.55	−144.98
Sum	−5464.11	−5028.81

**Table 3:** Individual BIC scores for the KL-control and expected utility models with estimated or fixed precision.

Subject	KL-control Estimated Precision	Expected Utility Estimated Precision	KL-control Fixed Precision	Expected Utility Fixed Precision
1	−239.29	−229.49	−255.92	−226.17
2	−293.71	−222.86	−309.05	−218.61
3	−348.59	−308.61	−445.15	−306.91
4	−170.69	−199.34	−172.67	−206.51
5	−203.75	−242.30	−202.28	−250.35
6	−325.17	−325.38	−339.77	−322.21
7	-369.97	−275.09	−511.98	−270.76
8	−157.99	−180.25	−189.65	−216.45
9	−207.68	−219.64	−202.67	−225.79
10	−180.67	−257.31	−217.53	−274.46
11	−239.47	−420.98	−250.11	−415.55
12	−271.68	−272.59	−273.76	−272.76
13	−184.31	−163.09	−188.14	−187.69
14	−159.99	−197.43	−189.95	−227.23
15	−208.62	−228.41	−203.39	−235.74
16	−178.65	−194.74	−197.41	−217.26
17	−376.28	−384.78	−399.06	−380.96
18	−173.00	−194.42	−196.05	−221.05
19	−228.63	−257.17	−224.27	−260.03
20	−161.95	−182.65	−202.54	−230.35
Sum	−4680.10	−4956.56	−5171.36	−5166.86

**Table 4:** Parameters of winning model and correlation with subject‘s rating.

Subject	Precision	Snack 1 (fixed)	Snack 2	Snack 3	Snack 4	Snack 5	Snack 6	Correlation
1	3.22	0	−3.11	1.18	−5.18	1.03	−2.53	0.78
2	2.71	0	−10.00	8.70	3.38	2.72	−9.99	0.70
3	4.42	0	5.23	2.85	−10.00	2.56	6.88	0.93
4	2.27	0	6.59	3.41	3.35	−10.00	6.26	0.95
5	2.39	0	−0.89	−4.63	0.05	−10.00	−1.66	0.96
6	2.00	0	0.13	4.12	−10.00	1.73	5.53	0.93
7	6.16	0	1.67	−4.84	−0.02	−6.22	1.12	0.38
8	5.39	0	−2.49	0.65	−0.73	−5.58	−3.59	0.93
9	2.10	0	−2.72	−10.00	-1.55	−0.97	−2.18	0.97
10	2.00	0	−0.86	−10.00	−1.16	−2.98	−2.19	0.94
11	8.00	0	−3.92	−2.71	5.26	0.89	−4.19	0.59
12	4.51	0	−3.04	−0.35	−2.77	−8.80	−2.44	0.86
13	8.00	0	−3.89	−3.57	-4.82	−2.35	−1.98	0.93
14	6.61	0	−0.02	0.02	0.01	0.42	0.03	0.95
15	2.70	0	1.19	2.95	3.00	−6.82	0.05	0.71
16	6.73	0	−7.37	−10.00	−0.54	3.56	−3.58	0.82
17	8.00	0	−3.82	−5.89	−1.61	−7.37	−5.64	0.96
18	4.39	0	−5.75	2.31	4.39	4.94	3.14	0.71
19	8.00	0	9.17	−10.00	2.07	2.62	3.77	0.98
20	2.00	0	0.67	−3.05	0.45	−0.39	0.24	0.97
